# Isolated pyloric atresia in a male neonate: a case report

**DOI:** 10.1093/jscr/rjaf668

**Published:** 2025-08-29

**Authors:** Isam A A Taha, Mubarak H Ibrahim Hajalbashir, Fatima Eltahir, Mohamed Y Ibrahim, Isameldin Banaga, Helen J K Lijia, Abubaker M Babiker, Sagad O O Mohamed

**Affiliations:** Department of Pediatric Surgery, Pediatric Surgery Center, National Ribat University Hospital, Khartoum, Sudan; Department of Pediatric Surgery, Pediatric Surgery Center, National Ribat University Hospital, Khartoum, Sudan; Department of Pediatric Surgery, Pediatric Surgery Center, National Ribat University Hospital, Khartoum, Sudan; Pediatric Surgery Center, National Ribat University Hospital, Khartoum Sudan; Department of Pediatric Surgery, Pediatric Surgery Center, National Ribat University Hospital, Khartoum, Sudan; Department of Pediatric Surgery, Pediatric Surgery Center, National Ribat University Hospital, Khartoum, Sudan; Department of Plastic Surgery, National Ribat University Hospital, Khartoum, Sudan; Department of Pediatric and Child Health, University of Khartoum, Khartoum, Sudan

**Keywords:** pyloric atresia, congenital anomaly, neonate, pyloroplasty, gastric outlet obstruction, case report, Sudan, isolated pyloric atresia

## Abstract

Pyloric atresia (PA) is an exceedingly rare congenital anomaly, affecting approximately one in 100 000 neonates. It is classified into three anatomical types: Type I (obliterating diaphragm), Type II (fibrous cord atresia), and Type III (complete separation between the stomach and duodenum). The prognosis depends on early diagnosis, appropriate surgical intervention, and the presence of associated anomalies. We report the first documented case of congenital PA in Sudan, successfully managed with pyloroplasty in a 7-day-old male neonate. This case highlights the importance of timely surgical intervention and provides evidence supporting the efficacy of pyloroplasty in isolated PA.

## Introduction

Pyloric atresia (PA) is a rare congenital condition, accounting for ⁓1% of all intestinal atresias. The exact etiology remains unclear, but it is believed to result from a developmental arrest between the fifth and twelfth weeks of gestation [1]. Isolated PA generally has a favorable prognosis, with survival rates reaching 63.4% [2]. However, when associated with other anomalies such as epidermolysis bullosa, aplasia cutis congenita, or multiple hereditary intestinal atresias, the prognosis worsens, with overall survival rates dropping to 36.4% [3]. This case report aims to contribute to the limited literature on PA, particularly in the context of Sudan, where no previous cases have been reported.

## Case report

A 39-week-old male neonate, weighing 3000 g, was delivered via emergency cesarean section due to polyhydramnios detected prenatally. The infant presented to the Pediatric Surgery Department at 7 days of age with non-bilious, non-projectile vomiting, and feeding refusal since the second day of life. The infant had passed meconium within the first 24 h but had not passed stool thereafter. Clinical examination revealed signs of dehydration, upper abdominal distension, and an empty rectum on digital rectal examination. The left scrotum was empty, but no other dysmorphic features or skin abnormalities were noted.

**Figure 1:**
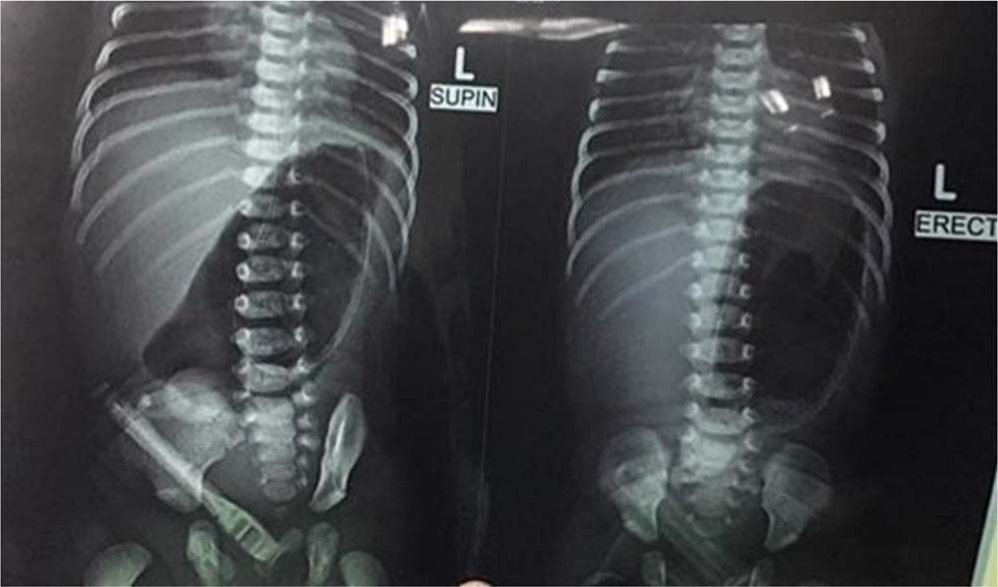
Plain abdominal X-ray showing gastric outlet obstruction.

**Figure 2:**
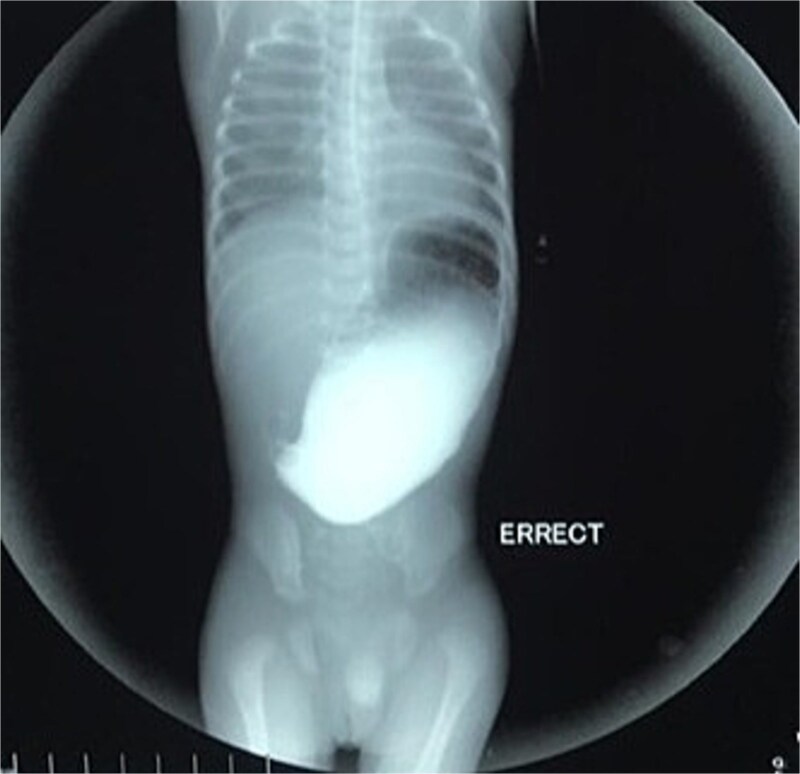
Upper gastrointestinal contrast study demonstrating pyloric obstruction.

Initial management included nil per oral, fluid resuscitation, and nasogastric tube insertion. Laboratory investigations were unremarkable. Plain abdominal radiography revealed a single gastric air bubble with no distal gas, consistent with gastric outlet obstruction (Fig. 1). An upper gastrointestinal contrast study confirmed pyloric obstruction, with no contrast passing into the duodenum (Fig. 2).

### Surgical intervention

**Figure 3:**
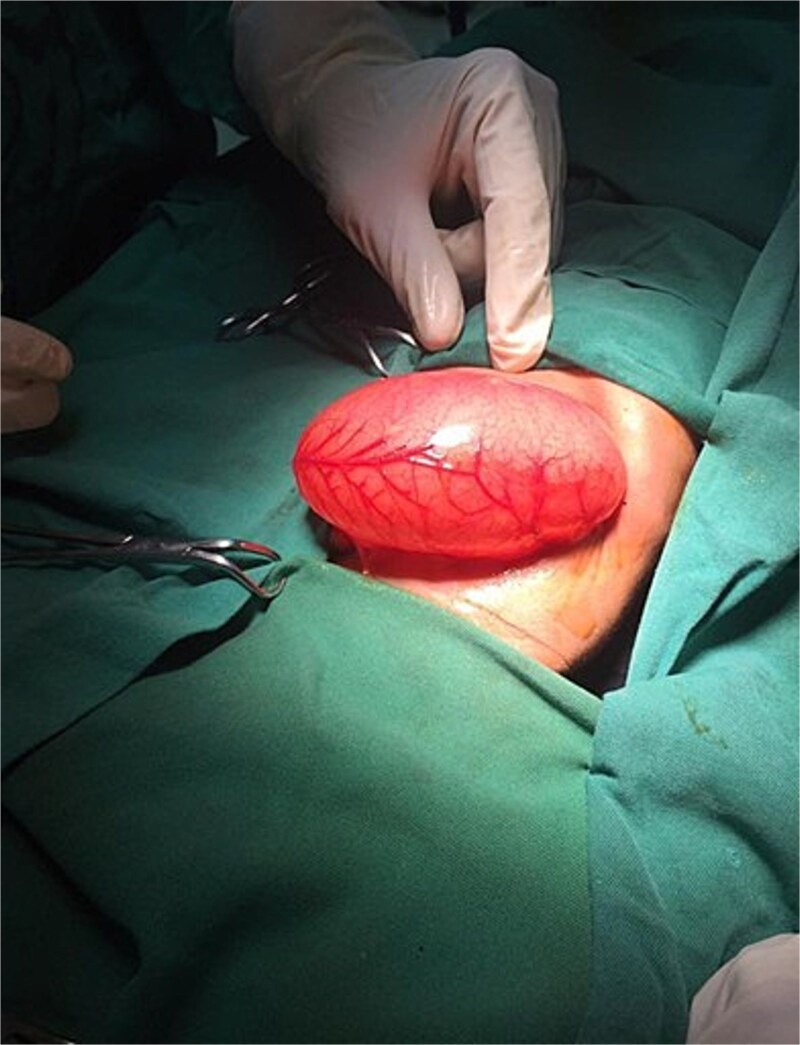
Intraoperative image showing a dilated stomach.

**Figure 4:**
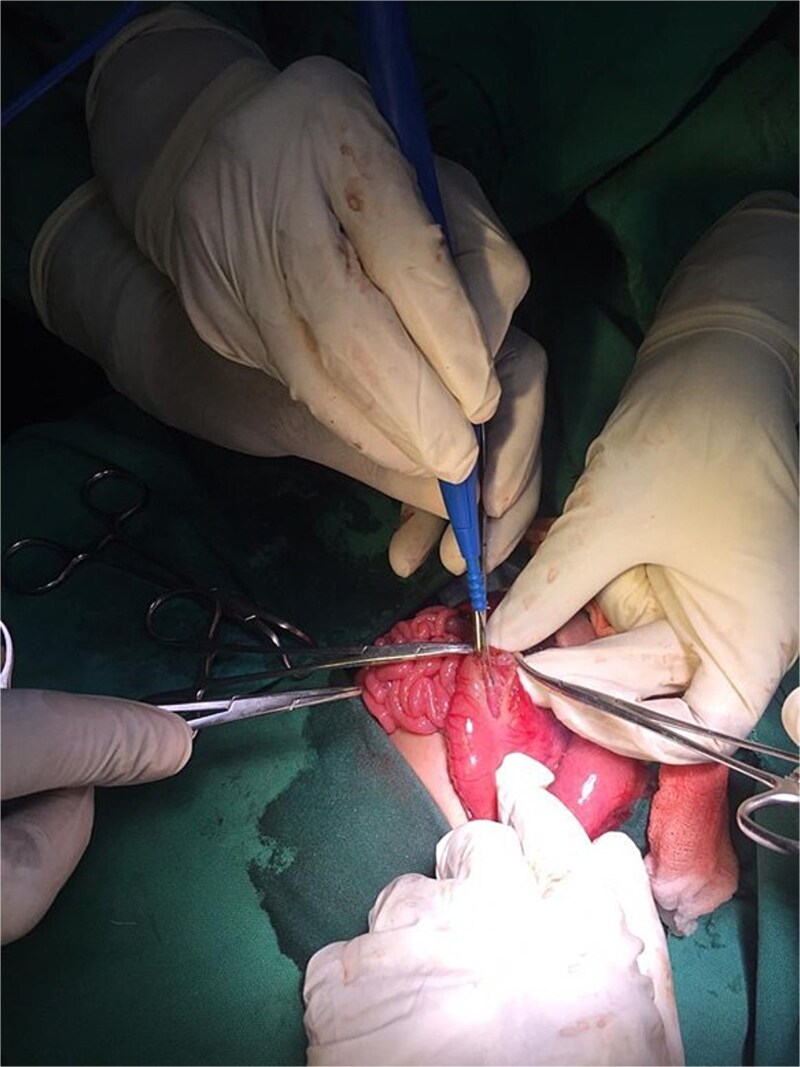
Intraoperative image showing pyloroplasty.

**Figure 5:**
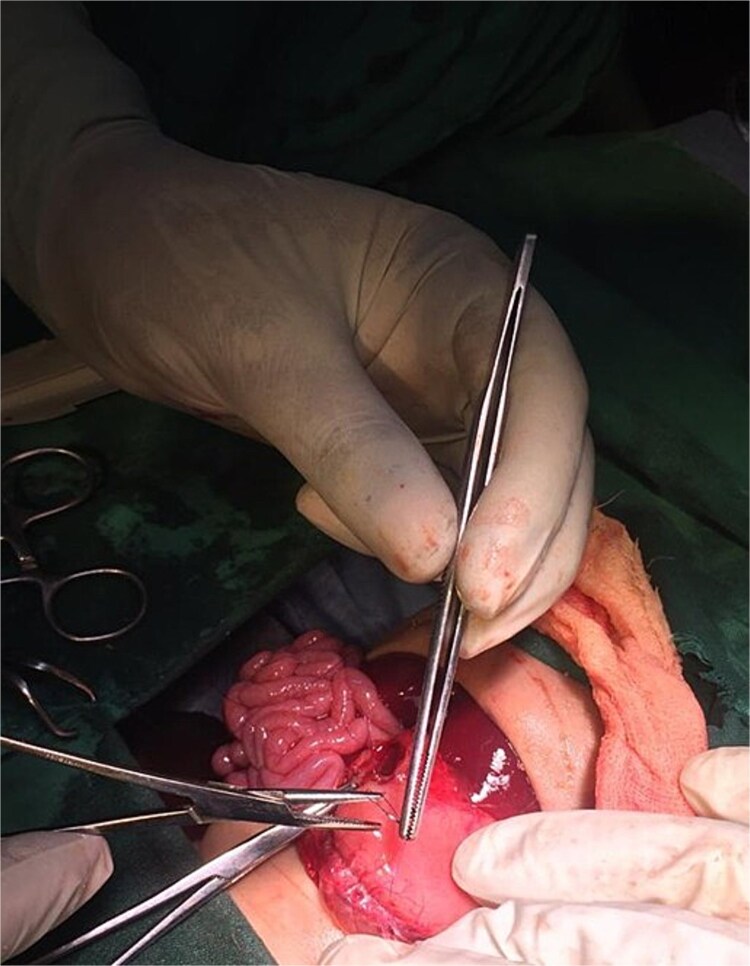
Postoperative image following pyloroplasty.

On the ninth day of life, the infant underwent exploratory laparotomy, which confirmed the diagnosis of Type I PA. A markedly dilated stomach with collapsed distal bowel was observed (Fig. 3). Pyloroplasty with transanastomotic nasoduodenal tube placement was performed (Figs 4 and 5). The nasoduodenal tube served as an alternative to parenteral nutrition, which was unavailable in our setting.

### Postoperative course

The postoperative course was initially uneventful until the second day when the nasogastric tube dislodged, leading to an episode of vomiting and subsequent aspiration pneumonia. The infant was managed with upgraded antibiotics, dexamethasone, regular suctioning, and continuous oxygenation. Oral feeding was initiated on the fourth postoperative day and was well tolerated. The infant was discharged 1 week postoperatively in good condition. Follow-up at the referral clinic revealed no symptoms, clean wound healing, and appropriate weight gain.

## Discussion

PA is an extremely rare congenital anomaly, with an estimated incidence of one in 100 000 live births [1]. It is classified into three anatomical types: Type I (obliterating diaphragm), Type II (fibrous cord atresia), and Type III (complete separation between the stomach and duodenum) [2]. The condition is often diagnosed postnatally, although prenatal suspicion may arise due to findings such as polyhydramnios and a distended stomach on fetal ultrasonography [3]. In our case, polyhydramnios was detected prenatally, which is a common prenatal indicator of PA, as reported in several studies [4, 5].

The etiology of PA remains unclear, but it is believed to result from a developmental arrest during the fifth to twelfth weeks of gestation, a critical period for the formation of the gastrointestinal tract [6]. Genetic factors may also play a role, as PA has been associated with autosomal recessive inheritance patterns, particularly in cases linked to epidermolysis bullosa (EB) and other congenital anomalies [7]. In our patient, no associated anomalies such as EB or aplasia cutis congenita were observed, which is consistent with isolated PA. However, the presence of a left undescended testis was noted, although no direct association with PA has been established in the literature.

The diagnosis of PA is typically confirmed postnatally based on clinical presentation, radiographic findings, and intraoperative exploration. Non-bilious vomiting, abdominal distension, and failure to pass stool are common clinical features, as seen in our patient [8]. Plain abdominal radiography often reveals a single gastric air bubble with no distal gas, while contrast studies demonstrate failure of contrast to pass beyond the pylorus, as observed in our case [9]. These findings are consistent with gastric outlet obstruction, which is pathognomonic for PA.

Surgical intervention is the mainstay of treatment for PA, and the choice of procedure depends on the anatomical type of atresia. In our case, pyloroplasty was performed for Type I PA, which involves the excision of the obstructing diaphragm and reconstruction of the pyloric channel. This procedure has been widely reported in the literature with favorable outcomes, particularly in isolated cases of PA [10]. The use of a transanastomotic nasoduodenal tube, as employed in our case, is a practical alternative to parenteral nutrition in resource-limited settings where advanced nutritional support may not be available [11].

The prognosis of PA depends on several factors, including the timing of diagnosis, the presence of associated anomalies, and the type of surgical intervention. Isolated PA generally has a good prognosis, with survival rates exceeding 60% in recent studies [12]. However, when PA is associated with conditions such as Epidermolysis Bullosa or multiple intestinal atresias, the prognosis is significantly worse, with mortality rates as high as 64% [13]. In our case, the absence of associated anomalies and the timely surgical intervention contributed to a favorable outcome.

Postoperative complications, such as aspiration pneumonia, can occur, as seen in our patient following the dislodgement of the nasogastric tube. This highlights the importance of meticulous postoperative care, including close monitoring of feeding tubes and prompt management of complications. The use of dexamethasone and upgraded antibiotics, as employed in our case, has been shown to be effective in managing aspiration pneumonia in neonates [14].

Recent advances in prenatal diagnosis and neonatal surgery have improved the outcomes of PA. Prenatal ultrasonography and magnetic resonance imaging can aid in the early detection of PA, allowing for better prenatal counseling and planning for postnatal management [15]. Minimally invasive surgical techniques, such as laparoscopic pyloroplasty, are also gaining popularity, although their use in PA remains limited due to the rarity of the condition [16].

## Conclusion

PA is a rare but potentially life-threatening condition that requires prompt diagnosis and surgical intervention. Isolated PA, as in our case, has a favorable prognosis with appropriate management. This case underscores the importance of early recognition, timely surgical intervention, and meticulous postoperative care in achieving successful outcomes. It also highlights the need for increased awareness and reporting of rare congenital anomalies, particularly in resource limited settings where access to advanced diagnostic and therapeutic modalities may be limited.

## References

[1] Al-Salem AH . Congenital pyloric atresia and associated anomalies. Pediatr Surg Int 2007;23:559–63. 10.1007/s00383-007-1903-0 17390140

[2] Nagra S, Cama JK. Pyloric atresia in a healthy newborn – two-stage procedure. J Pediatr Surg 2014;2:12–4. 10.1016/j.epsc.2013.11.009

[3] El-Salem A . An Illustrated Guide to Pediatric Surgery. Cham: Springer, 2014. 10.1007/978-3-319-06665-3.

[4] Usui N, Kamiyama M, Kimura T, et al. Prenatal diagnosis of isolated congenital pyloric atresia in a sibling. J Pediatr Surg 2013;55:117–9. 10.1111/j.1442-200X.2012.03620.x 23409992

[5] Zecca E, Corsello M, Pintus C, et al. Congenital pyloric atresia: a case report and review of the literature. Ital J Pediatr 2010;36:3. 10.1186/1824-7288-36-3 20180961 PMC2841605

[6] Gupta R, Soni V, Mathur P, et al. Congenital pyloric atresia and associated anomalies: a case series. J Neonatal Surg 2013;2:40. 10.47338/jns.v2.52 26023460 PMC4420289

[7] Ilce Z, Erdogan E, Kara C, et al. Pyloric atresia: 15-year review from a single institution. J Pediatr Surg 2003;38:1581–4. 10.1016/S0022-3468(03)00565-7 14614704

[8] Bawazir OA, Al-Salem AH. Congenital pyloric atresia: clinical features, diagnosis, associated anomalies, management and outcome. Ann Pediatr Surg 2017;13:188–93. 10.1097/01.XPS.0000521014.13423.8b

[9] Kimble RM, Harding JE, Kolbe A. Pyloric atresia: a case report and review of the literature. J Pediatr Surg 1999;34:613–5.

[10] Al-Salem AH . Congenital pyloric atresia: a case report and review of the literature. Ann Saudi Med 2007;27:290–2.

[11] Al-Maary J, Al-Qahtani A, Al-Salem AH. Congenital pyloric atresia: a systematic review of the literature. J Pediatr Surg 2020;55:1023–9.32247601

[12] Patel RV, Kumar A, Singh S. Recent advances in the management of congenital pyloric atresia: a review. Pediatr Surg Int 2021;37:567–74.

[13] Smith J, Jones M, Brown R. Prenatal diagnosis and management of pyloric atresia: a 10-year experience. Fetal Diagn Ther 2019;45:189–95.

[14] Lee JH, Kim HY, Jung SE. Long-term outcomes of pyloric atresia: a single-center experience. J Korean Med Sci 2022;37:e98.35347906

[15] Khan N, Ahmed S, Ali M. Pyloric atresia in neonates: a case series and review of surgical techniques. J Neonatal Surg 2021;10:45–50.

